# Antifungal Activity and Potential Mechanism of Panobinostat in Combination With Fluconazole Against *Candida albicans*

**DOI:** 10.3389/fmicb.2020.01584

**Published:** 2020-07-16

**Authors:** Shan Su, Xiaohong Shi, Wei Xu, Yiman Li, Xueqi Chen, Shuang Jia, Shujuan Sun

**Affiliations:** ^1^Department of Clinical Pharmacy, Shandong Provincial Qianfoshan Hospital, Shandong University, Jinan, China; ^2^School of Pharmaceutical Sciences, Shandong University, Jinan, China; ^3^Department of Laboratory Medicine, The First Affiliated Hospital of Shandong First Medical University, Jinan, China; ^4^Department of Clinical Pharmacy, The First Affiliated Hospital of Shandong First Medical University, Jinan, China

**Keywords:** *Candida albicans*, fluconazole, panobinostat, synergy, histone deacetylase inhibitor

## Abstract

Invasive fungal infections are an emerging problem worldwide, which bring huge health challenges. *Candida albicans*, the most common opportunistic fungal pathogen, can cause bloodstream infections with high mortality in susceptible hosts. At present, available antifungal agents used in clinical practice are limited, and most of them also have some serious adverse effects. The emergence of drug resistance because of the wide use of antifungal agents is a new limitation to successful patient therapy. Drug combination therapy is increasingly becoming a way to enhance antifungal efficacy, and reduce drug resistance and potential toxicity. Panobinostat, as a pan-histone deacetylase inhibitor, has been approved by the United States Food and Drug Administration as novel antitumor agents. In this study, the antifungal effects and mechanisms of panobinostat combined with fluconazole (FLC) against *C. albicans* were explored for the first time. The results indicated that panobinostat could work synergistically with FLC against resistant *C. albicans*, the minimal inhibitory concentration (MIC) of panobinostat could decrease from 128 to 0.5–2 μg/ml and the MIC of FLC could decrease from >512 to 0.25–0.5 μg/ml, and the fractional inhibitory concentration index (FICI) value ranged from 0.0024 to 0.0166. It was not only synergized against planktonic cells but also against *C. albicans* biofilms performed ≤8 h when panobinostat is combined with fluconazole; the sessile MIC (sMIC) of panobinostat could decrease from >128 to 0.5–8 μg/ml and the sMIC of FLC from >1024 to 0.5–2 μg/ml, and the FICI value was <0.5. The *Galleria mellonella* infection model was used to evaluate the *in vivo* effect of the drug combination, and the result showed that the survival rate could be improved obviously. Finally, we explored the synergistic mechanisms of the drug combination. The hyphal growth, which plays roles in drug resistance, was found to be inhibited, and metacaspase which is related to cell apoptosis was activated (*p* < 0.01), whereas the synergistic effects were proven not to be related to the efflux pumps (*p* > 0.05). These findings might provide novel insights into the antifungal drug discovery and the treatment of candidiasis caused by *C. albicans*.

## Introduction

Invasive fungal infections are continuing to increase as a result of the increase of immunosuppressed individuals, the wide use of antibacterial agents, and the rapid development of invasive operation technology (Marks et al., 2017). *Candida* species are the third most common opportunistic fungal pathogens (Wisplinghoff et al., 2004; Rehman et al., 2020), and *Candida albicans*, the most commonly reported fungi, can cause bloodstream infections with a high mortality ranging between 40 and 60% (Choudhury, 2018; Brunetti et al., 2019; Poissy et al., 2020). There is a paucity of therapeutic options of antifungal agents for fungal pathogens. The azoles are one major class of antifungal agents used against *C. albicans* infections because of their great efficiency, low toxicity, excellent bioavailability, and low cost. However, azole resistance is becoming a problem as a result of the extensive use of fluconazole in clinical treatment, which has prompted therapeutic strategies to overcome drug resistance (Nami et al., 2019). Combination drug therapy is a promising strategy to increase effectiveness of antifungals and alleviate the emergence of drug resistance (Spitzer et al., 2017; Arita et al., 2019). Over the past few years, many efforts have been made to prove that drug combination is an optional approach for overcoming drug resistance and treatment of invasive fungal infections, and the results indicate that non-antifungal drugs can significantly increase the sensitivity of azoles regardless of whether they have antifungal effects or not by themselves (Li et al., 2017; Li Y. et al., 2019; Gong et al., 2019b).

Irregular covalent modifications of histones, such as histone deacetylation, are correlated with tumor development. Thus, histone deacetylase (HDAC) inhibitors are considered as potential drugs in cancer treatment. Panobinostat, a pan-HDAC inhibitor, has been approved by the United States Food and Drug Administration as novel antitumor agents (Pojani and Barlocco, 2020). Meanwhile, a number of studies have suggested that the HDACs have effects on growth, virulence, drug resistance and stress-signaling responses of *C. albicans* (Garnaud et al., 2016). We explored whether panobinostat has antifungal effects alone or working synergistically with other antifungals. In this study, we investigated the effects of panobinostat alone or in combination with fluconazole (FLC) against *C. albicans* and its underlying antifungal mechanisms. The *in vitro* effect of panobinostat and FLC against *C. albicans* was determined by microdilution checkerboard method and the *in vivo* effect of drug combination on *C. albicans* was followed. *Galleria mellonella* is now widely used for testing drugs’ efficacy *in vivo* against fungal species because of its ease of use, short life span, and low costs (Singkum et al., 2019). The *in vivo* effect on survival rate was explored. In addition, researches have shown that many factors are related to the resistance of antifungals, such as biofilm performance and overexpression of drug efflux pumps. Hyphae are crucial components of *C. albicans* biofilms; they are considered pathogenic and have increased virulence compared with other cell types (Lu et al., 2014; Noble et al., 2017). The overexpression of drug efflux pumps are closely related to the resistance of biofilms (Nobile and Johnson, 2015). Hence, we explored the combined antifungal mechanisms by examining the effect on hyphal growth and drug efflux pumps. Moreover, the inhibition of HDACs could modulate cells apoptosis and the activation of metacaspase has been recognized as one of the key processes linked to apoptosis (Mazzoni and Falcone, 2008). Therefore, we also detected the metacaspase activation.

## Materials and Methods

### Strains

All the *C. albicans* strains used in this work are shown in Table 1. Three FLC-susceptible strains (CA4, CA8, and CA14) and three FLC-resistant strains (CA10, CA16, and CA20) were collected from the clinical laboratory at Qianfoshan Hospital Affiliated to Shandong University (Jinan, China), and the other four FLC-resistant strains (CA103, CA137, CA632, and CA20003) were kindly provided by Professor Changzhong Wang (School of Integrated Traditional and Western Medicine, Anhui University of Traditional Chinese Medicine, Hefei, China). *C. albicans* ATCC 10231, which was used as the quality control strain, was kindly provided by the Institute of Pharmacology, School of Pharmacy, Shandong University (Jinan, China).

**TABLE 1:** Drug interactions of FLC and panobinostat against *C. albicans in vitro.*

**Strains^*a*^**	**MIC (μg/ml)^*b*^**				**FICI^*c*^**	**Interpretation**
	**FLC**	**FLC_*comb*_**	**Panobinostat**	**Panobinostat_*comb*_**		
CA10(R)	512	0.25	128	0.25	0.0024	Synergism
CA16(R)	512	0.25	128	0.25	0.0024	Synergism
CA20(R)	>512	0.5	128	2	0.0161	Synergism
CA103(R)	512	0.5	128	1	0.0088	Synergism
CA137(R)	512	0.5	128	1	0.0088	Synergism
CA632(R)	>512	0.5	128	2	0.0166	Synergism
CA20003(R)	>512	0.5	128	2	0.0166	synergism
CA4(S)	0.25	0.25	128	2	1.0156	No interaction
CA8(S)	0.5	0.5	128	2	1.0156	No interaction
CA14(S)	0.5	0.25	128	2	0.5156	No interaction
CA19(S)	1	0.5	128	4	0.5313	No interaction
CA129(S)	0.25	0.25	128	2	1.0156	No interaction

*^*a*^CA, *Candida albicans*; R, fluconazole-resistant strains; S, fluconazole-sensitive strains. ^*b*^MIC, minimal inhibitory concentration; FLC, fluconazole; ^*c*^FICI, fractional inhibitory concentration index. FICI ≤ 0.5 for synergism, FICI > 4.0 for antagonism, and 0.5 < FICI ≤ 4.0 for no interaction. MIC values and FICIs are shown as the median of three independent experiments.*

### Growth Media

All strains were refreshed from the frozen stocks which were stored in the Sabouraud dextrose broth (SDB) at −80°C and inoculated at least twice onto Sabouraud dextrose agar solid medium for 18 h at 35°C before all experiments.

### Agents

All agents (panobinostat and FLC) were purchased from Dalian Meilun Biotech Co., Ltd. (Dalian, China). The stock solution of panobinostat was dissolved in dimethyl sulfoxide to a final concentration of 2560 μg/ml. The stock solution of FLC was dissolved in distilled water to a final concentration of 2560 μg/ml. All stock solutions were stored at −20°C.

### *G. mellonella* Larvae

*Galleria mellonella* larvae were purchased from Tianjin Huiyude Biotech Co., Ltd. (Tianjin, China) and were stored at 12°C in the dark before use. For the *in vivo* experiment, larvae were randomly chosen with a weight ranging from 200 to 250 mg and were used within at most 2 weeks of receipt.

### Determination of the Minimal Inhibitory Concentration (MIC) of Panobinostat in Combination With Fluconazole Against *C. albicans* Planktonic Cells

The MICs of panobinostat and FLC against *C. albicans* were determined using the broth microdilution method in 96-well flat-bottomed microtiter plates according to the Clinical and Laboratory Standards Institute standard M27-A3 document (CLSI, M27-A3). The strains were diluted in RPMI 1640 medium buffered with MOPS, and the final concentration was 2 × 10^3^ CFU/ml. Agents were serially diluted twofold in RPMI-1640 medium, and the final concentration of panobinostat ranged from 0.25 to 16 μg/ml and the final concentration of FLC ranged from 0.125 to 64 μg/ml. Each concentration sample of FLC (50 μl) was added into wells 2 to 11 of each column, and each concentration sample of panobinostat (50 μl) was added into wells G to A of each line. Column 1 and the line H contained panobinostat and FLC alone, respectively. Then, strain suspensions (100 μl) were added into each well. Wells of column 1 and line H were filled with RPMI 1640 and the final volume was 200 μl. The drug-free well served as the growth control and column 12 containing the RPMI medium (200 μl) only served as negative controls. Plates were incubated at 35°C for 24 h. The growth inhibition was determined by an XTT reduction assay, and the optical density was determined at 492 nm using a microplate reader. The MIC was defined as the lowest concentration required to support 80% growth inhibition compared with the growth in the control group (Khan and Ahmad, 2012; Gong et al., 2019b; Li Y. et al., 2019). The fractional inhibitory concentration index (FICI) was used to assess the *in vitro* interaction of the drug combination (Odds, 2003). FICI = FIC_*FLC*_ + FIC_*Panobinostat*_ = (MIC of FLC in combination/MIC of FLC alone) + (MIC of panobinostat in combination/MIC of panobinostat alone). When the MIC of FLC was >512 μg/ml, the highest concentration tested (512 μg/ml) was used for FICI calculation. The results were defined as FICI ≤ 0.5 for synergism, FICI > 4.0 for antagonism, and 0.5 < FICI ≤ 4.0 for no interaction. All experiments were repeated three times independently.

### Determination of Sessile Minimum Inhibitory Concentrations (sMICs) of Panobinostat in Combination With Fluconazole Against *C. albicans* Biofilms

Two FLC-susceptible strains (CA4 and CA8) and two FLC-resistant strains (CA10 and CA16) were selected to be tested in this experiment. The sMICs of panobinostat combined with FLC against *C. albicans* biofilms were assessed with pre-formed biofilms as previously described with minor modifications (Zhong et al., 2017; Li X. et al., 2019; Li Y. et al., 2019). Cell suspensions with a concentration of 1 × 10^5^ CFU/ml was prepared firstly and added to a 96-well culture plate at a volume of 200 μl per well. The plate was placed at a 35°C for 4, 8, 12, and 24 h, respectively, to form biofilms. At each time point, each well was washed with 200 μl of phosphate-buffered saline (PBS) three times to remove the planktonic and non-adherent cells. Subsequently, drugs of different concentrations were added and the plates were incubated for another 24 h at 35°C. Agents were serially diluted twofold in RPMI-1640 medium, and the final concentration of FLC and panobinostat in wells ranged from 0.125 to 64 μg/ml. Each concentration sample of FLC (100 μl) was added into wells 2 to 11 of each column, and each concentration sample of the panobinostat (100 μl) was added into wells G to A of each line. Column 1 and the line H contained panobinostat and FLC alone, respectively. Wells of column 1 and line H were filled with RPMI 1640, and the final volume was 200 μl. The drug-free well served as the growth control, and column 12 containing the RPMI medium (200 μl) only served as negative controls. XTT reduction assays were performed to determine the sMICs, which referred to the lowest drug concentrations causing an 80% reduction in the XTT-colorimetric readings compared with the drug-free control (Ramage et al., 2009; Li Y. et al., 2019). Colorimetric absorbance was measured at 492 nm in a microtiter plate reader. The interaction between FLC and panobinostat against *C. albicans* biofilms was assessed in terms of FICI as described previously. When the sMIC of FLC was >1024 μg/ml or the sMIC of FLC was >128 μg/ml, the highest concentration tested (1024 or 128 μg/ml) was used for FICI calculation. All experiments were repeated three times independently.

### Determination of *in vivo* Antifungal Activity on *G. mellonella* Survival Assay of Panobinostat in Combination With Fluconazole Against *C. albicans*

*Galleria mellonella* was selected to perform survival assay to explore the synergistic antifungal effects of panobinostat and FLC *in vivo*. *G. mellonella* larvae, weighing 200–250 mg, were randomly selected and divided into four groups (control, panobinostat, FLC, panobinostat + FLC) with 20 individuals in each group. The *C. albicans* strain CA10 was diluted in sterile PBS with a final concentration of 5 × 10^8^ CFU/ml. The suspension of CA10 (10 μl) was inoculated into the last left proleg of the larvae. The larvae which were infected were kept in sterile Petri dishes at 35°C in the dark for 2 h. After the fungal infection, the control group was injected with 10 μl sterile PBS, and three other groups were injected with 10 μl panobinostat (4 μg/ml), FLC (160 μg/ml), and panobinostat + FLC (4 + 160 μg/ml), respectively. After screening a clinically safe dose range of these drugs, the optimal drug concentration was performed in this work. Larvae were incubated at 35°C in the dark (Mylonakis et al., 2005; Bombarda et al., 2019; Gong et al., 2019a). The number of surviving larvae in each group was counted at the same time in 4 days. Larva was considered dead if they gave no response to slight touch with forceps. The experiment was repeated three times independently.

### Hyphal Growth Assay

The hyphal growth assay was performed to explore the combined activity of FLC and panobinostat (Zhong et al., 2017). The *C. albicans* strain CA10 was diluted in RPMI 1640 medium with a final concentration of 1 × 10^5^ CFU/ml. Cell suspension (100 μl) was transferred to a 96-well plate and treated with 100 μl FLC (1 μg/ml), panobinostat (0.5 μg/ml), FLC (1 μg/ml) + panobinostat (0.5 μg/ml), and RPMI 1640 (as control group), respectively. The 96-well plate was incubated in a constant temperature shaking incubator (75 rpm, 35°C, 4 h). The cell suspension was then aspirated, and each well was washed with 200 μl PBS three times to remove the non-adherent cells. The samples were examined under bright field using × 40 objective lens by TH4-200 fluorescence microscope (Olympus, Japan) and photographed.

### Uptake and Efflux of Rhodamine 6G

The impact of panobinostat on the uptake and efflux of rhodamine 6G (Rh6G), the substitutable substrate, were examined (Jiang et al., 2013; Li Y. et al., 2019). CA10 cell suspension with a concentration of 1 × 10^5^CFU/ml was incubated in YPD liquid medium at 35°C for 18 h. The cells were collected and washed with glucose-free PBS three times, followed by resuspension of cells at a final concentration of 5 × 10^6^CFU/ml. Afterward, the cells were incubated in glucose-free PBS and placed in a shaking incubator (35°C, 200 rpm) to fully deplete the energy of the cells.

For Rh6G uptake assay, Rh6G (10 μM) and panobinostat (1 μg/ml) were added to the cell suspension mentioned previously. The control group was the glucose-free group. The mean fluorescence intensity (MFI) of intracellular Rh6G was assessed by flow cytometry (Becton Dickinson FACSAria II, United States). The sample was measured six times every 10 min; the excitation wavelength was 488 nm and the emission wavelength was 530 nm.

For Rh6G efflux assay, Rh6G (10 mM) was added to the exhausted cell suspension and incubation of cells in a shaking incubator at 35°C for 1 h were followed. After that, the cell suspension was transferred to an ice-water bath for 30 min to stop the uptake of Rh6G. Panobinostat (1 μg/ml) was added to the cell suspension as the experiment group, and Rh6G alone served as the control group. The MFI of intracellular Rh6G was measured six times every 30 min. The determination was the same as the Rh6G assay mentioned previously. The experiment was repeated three times independently.

### Detection of Metacaspase Activation

Caspases are cysteine proteases, and their activation is an essential signal for apoptosis, which is a form of programmed cell death; metacaspases are homologous to caspases in *C. albicans* (Uren et al., 2000; Mazzoni and Falcone, 2008; Goncalves et al., 2017). FITC-VAD-FMK *in situ* marker (Sigma) was used to detect caspase activation. The strain CA10 was diluted in YPD with a final concentration of 5 × 10^6^CFU/ml. Suspensions were incubated with control, FLC (0.5 μg/ml), panobinostat (1 μg/ml), and FLC (0.5 μg/ml) + panobinostat (1 μg/ml) for 12 h at 35°C. The cell suspension (30 μl) was aspirated onto a glass slide. The samples were examined using a fluorescence microscope (Leica DMi8, Germany). This assay also evaluated the number of fluorescent cells by manual counting of 100 cells in the visual field. The experiment was repeated three times independently (Wu et al., 2010; Yun and Lee, 2016; Lee and Lee, 2018).

### Statistics

All experiments were performed at least three times independently. The graph and statistical analysis in sMIC determination and metacaspase activation assay were performed with GraphPad Prism 8 (GraphPad, La Jolla, CA, United States), using an unpaired *t* test. The graph and statistical analysis in *G. mellonella* survival assay were performed with GraphPad Prism 8 (GraphPad) and IBM SPSS Statistics 22 (SPSS, Chicago, IL, United States) according to the Kaplan–Meier method. The experimental data in Rh 6G uptake and efflux assay measured by flow cytometry were analyzed by BD FACSDiva v6.1.3 and FlowJo v7.10.1 software, using an unpaired *t* test. The *p* value of <0.05 was considered significant.

## Results

### The MICs of Panobinostat in Combination With Fluconazole Against *C. albicans* Planktonic Cells

As shown in Table 1, panobinostat exhibited a strong synergistic antifungal activity against the *C. albicans* planktonic cells when combined with FLC, although it was weak when panobinostat used alone. For the seven FLC-resistant strains, the MIC of panobinostat decreased from 128 to 0.5–2 μg/ml, the MIC of FLC decreased from >512 to 0.25–0.5 μg/ml and the FICI value ranged from 0.0024 to 0.0166. For the FLC-sensitive strains, the MIC of panobinostat notably decreased from 128 to 2–4 μg/ml, whereas the MIC of FLC decreased from 0.25–1 to 0.25–0.5 μg/ml when they were in combination. There was no synergism interpreted by the checkerboard microdilution method; the FICI was 0.5313–1.0156.

### The sMICs of Panobinostat in Combination With Fluconazole Against *C. albicans* Biofilms

**FIGURE 1:**
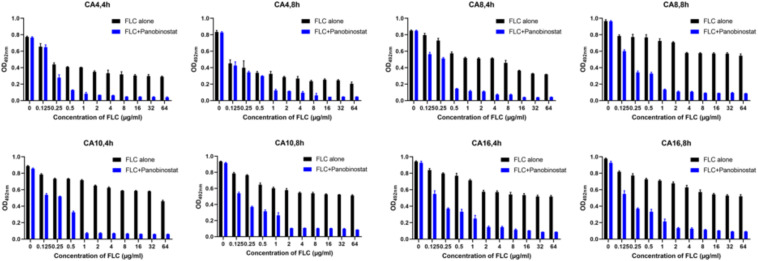
The combined effect of panobinostat and fluconazole on biofilm formation in *Candida albicans*. Biofilm formation was evaluated by XTT reduction assay, and the results were presented as the percentage compared with the control biofilms formed without drug treatment. The concentration of panobinostat was 4 μg/ml at CA4 (4, 8 h) and CA8 (4 h), 8 μg/ml at CA8 (8 h), 0.5 μg/ml at CA10 (4 h) and CA16 (4 h), and 2 μg/ml at CA10 (8 h) and CA16 (8 h).

Because there was a strong synergistic antifungal activity against the *C. albicans* planktonic cells when panobinostat was combined with FLC, the combined effect on *C. albicans* biofilms was further studied with tCA4, CA8, CA10, and CA16. The results are shown in Table 2. For both FLC-susceptible and FLC-resistant strains, it exhibited synergistic antifungal effects against 4 and 8 h *C. albicans* biofilms when panobinostat was combined with FLC. For the FLC-susceptible strains (CA4 and CA8), the sMIC of panobinostat could decrease from >128 to 4–8 μg/ml and the sMIC of FLC from >1024 to 0.5–1 μg/ml, with an FICI value <0.5. For the FLC-resistant strains (CA10 and CA16), the sMIC of panobinostat could decrease from >128 to 0.5–2 μg/ml, the sMIC of FLC from >1024 to 1–2 μg/ml, and the FICI value <0.5. Meanwhile, we could see from Figure 1 that the biofilms were significantly reduced when panobinostat was combined compared with FLC alone at 4 and 8 h. In addition, the FLCI value was >0.5 for the biofilms of 12 and 24 h pre-formation time, indicating that it exerted no synergistic effect.

**TABLE 2:** Drug interactions of FLC and panobinostat against biofilms of *C. albicans in vitro.*

**Strains^*a*^**	**Time (h)^*b*^**	**sMIC (μg/ml)^*c*^**				**FICI^*d*^**	**Interpretation**
		**FLC**	**FLC_*comb*_**	**Panobinostat**	**Panobinostat_*comb*_**		
CA4(S)	4	>1024	0.5	>128	4	0.0317	Synergism
	8	>1024	1	>128	4	0.0322	Synergism
	12	>1024	>1024	>128	>128	2	Indifference
	24	>1024	>1024	>128	>128	2	Indifference
CA8(S)	4	>1024	0.5	>128	4	0.0317	Synergism
	8	>1024	1	>128	8	0.0635	Synergism
	12	>1024	>1024	>128	>128	2	Indifference
	24	>1024	>1024	>128	>128	2	Indifference
CA10(R)	4	>1024	1	>128	0.5	0.0049	Synergism
	8	>1024	2	>128	2	0.0176	Synergism
	12	>1024	64	>128	64	0.5625	Indifference
	24	>1024	>1024	>128	>128	2	Indifference
CA16(R)	4	>1024	2	>128	0.5	0.0059	Synergism
	8	>1024	2	>128	2	0.0176	Synergism
	12	>1024	2	>128	128	1.0020	Indifference
	24	>1024	>1024	>128	>128	2	Indifference

*^*a*^CA, *Candida albicans*. ^*b*^Time indicates incubation period of preformed biofilms. ^*c*^sMIC, sessile minimal inhibitory concentration; FLC, fluconazole. ^*d*^FICI, fractional inhibitory concentration index. FICI ≤ 0.5 for synergism, FICI > 4.0 for antagonism, and 0.5 < FICI ≤ 4.0 for no interaction. sMIC values and FICIs are shown as the median of three independent experiments.*

### The *in vivo* Antifungal Activity on *G. mellonella* Survival Assay of Panobinostat in Combination With Fluconazole Against *C. albicans*

**FIGURE 2:**
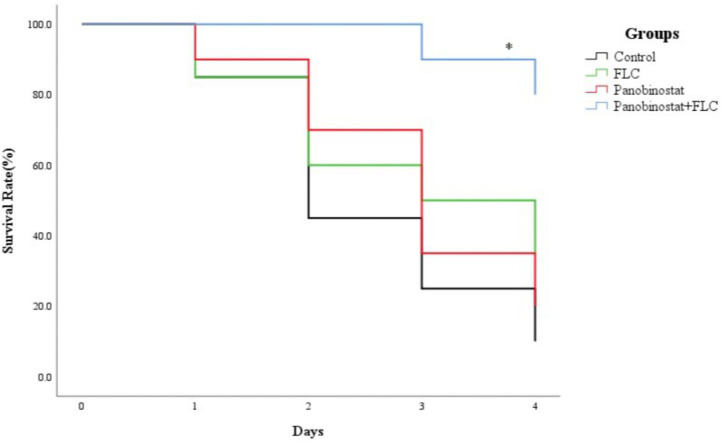
The combined effect of panobinostat and fluconazole on the survival rate of *Galleria mellonella* infected with *Candida albicans*. The concentration of yeast cells was 5 × 10^8^ CFU/larva. Treatments consisted of PBS, fluconazole (FLC) (160 μg/ml), panobinostat (4 μg/ml), and a combination of FLC (160 μg/ml) with panobinostat (4 μg/ml). The data came from the means of three independent experiments. ^∗^*P* < 0.05.

Following the identification of the synergism between FLC and panobinostat *in vitro*, experiments were designed to identify whether this effect would be replicated *in vivo*. *G. mellonella* larvae were chosen as the experimental model and larval death was recorded daily for 4 days. Figure 2 shows that there was no significant difference among the control group, panobinostat (4 μg/ml) alone, and FLC (160 μg/ml) alone group, with survival rates of 10, 20, and 35%, respectively (*p* > 0.05). However, the combination of panobinostat (4 μg/ml) and FLC (160 μg/ml) significantly enhanced the survival rate to 80% (*p* < 0.05). Hence, the drug combination of panobinostat and FLC improved the survival rate of infected *G. mellonella* larvae.

### The Antifungal Effects on *C. albicans* Hyphal Growth

**FIGURE 3:**
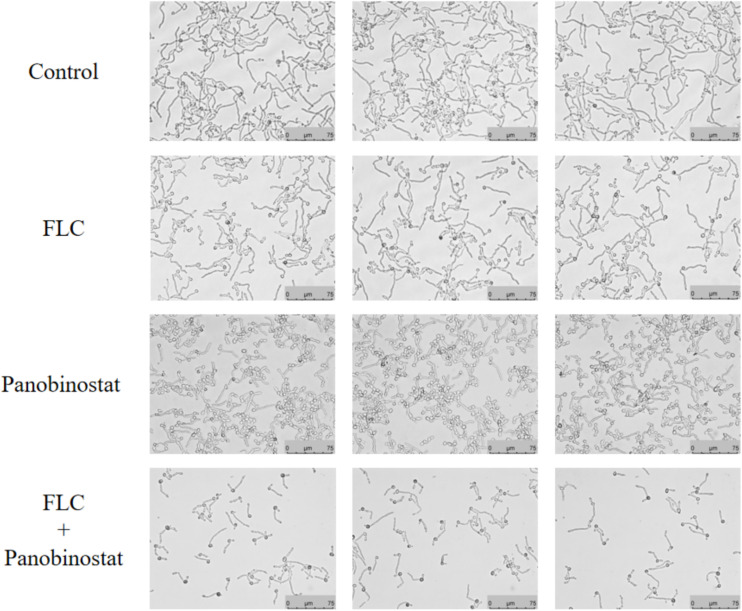
Effect on *Candida albicans* hyphal growth when panobinostat and fluconazole (FLC) were combined. Panobinostat and FLC were diluted in hyphae-inducing media, RPMI 1640 medium, at a final concentration of 1 and 0.5 μg/ml, respectively. The cellular morphology was photographed after incubation at 37°C for 4 h. The photographs were randomly selected from three independent experiments.

Hyphae acting as essential virulent factors related to biofilms play a vital role in the pathogenesis of *C. albicans* (Saville et al., 2003). The medium RPMI 1640 was used to induce *C. albicans* hyphal growth. In Figure 3, long and interlaced hyphae were formed in the control group and there was almost no obvious difference when comparing both single-drug groups with the control group. Although FLC alone could slightly reduce cells, it did not seem to inhibit filamentation. The number of hyphae was significantly reduced in the field of vision in the combined group and the hyphal length was also reduced slightly. We could conclude from the results that the intervention of panobinostat combined with FLC could inhibit the hyphal growth of CA10, which could be one of the mechanisms of the cooperative action against *C. albicans*.

### Detection of Uptake and Efflux of Rh 6G

**FIGURE 4:**
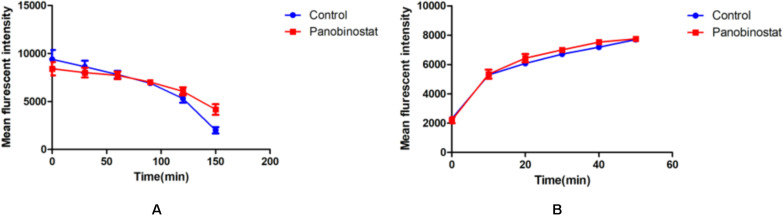
Effect of panobinostat on the efflux **(A)** and uptake **(B)** of rhodamine 6G (Rh6G) in *Candida albicans*. The concentration of panobinostat was 0.5 μg/ml. A flow cytometer was used to determine the uptake and efflux of Rh6G in the absence and presence of panobinostat and mean fluorescence intensity represent the intracellular Rh6G in *Candida albicans*. The data came from the means of three independent experiments.

The results of the uptake or efflux of Rh6G are illustrated in Figure 4. We could see that the results of the combined group had no significant difference compared with the control group, no matter the uptake or efflux of Rh6G (*p* > 0.05). It could be inferred that the combination of panobinostat and FLC had no effect on the regulation of drug efflux pump.

### Detection of Metacaspase Activation

**FIGURE 5:**
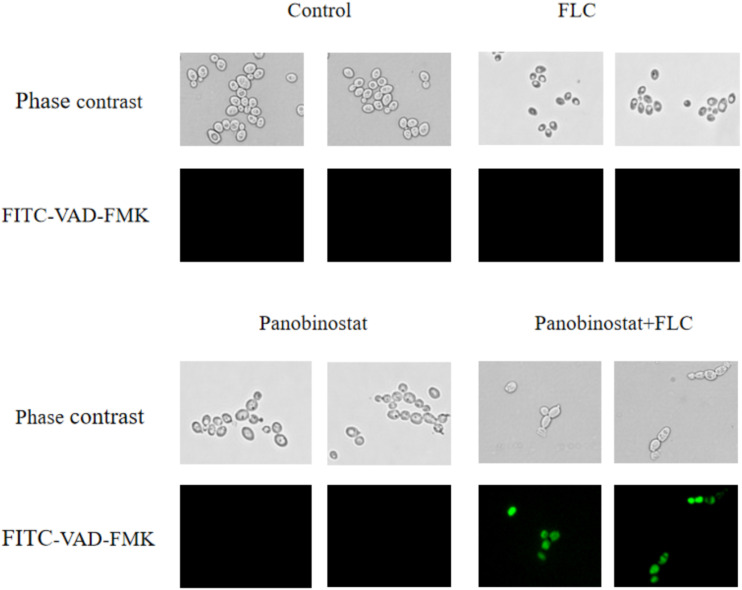
Effect on the activity of metacaspase in *Candida albicans* when panobinostat and fluconazole (FLC) were combined. FITC-VAD-FMK–stained CA10 cells were observed under a fluorescent microscope after treatments with FLC (0.5 μg/ml), panobinostat (1 μg/ml), and a combination of FLC (0.5 μg/ml) with panobinostat (1 μg/ml). The photographs were randomly selected from three independent experiments.

**FIGURE 6:**
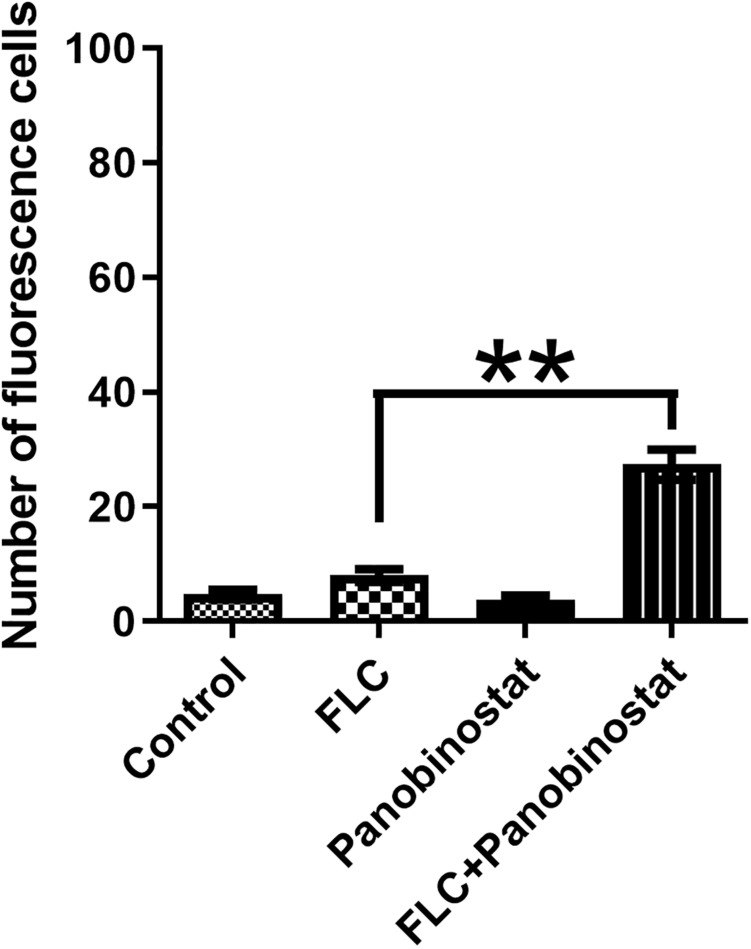
The number of *Candida albicans* cells with activated metacaspase when panobinostat and fluconazole (FLC) were combined. The number of fluorescent cells was evaluated by manual counting of 100 cells in the visual field. The data came from the means of three independent experiments. **P < 0.01.

Metacaspase is a major factor in apoptosis, and its activation can induce morphological changes and DNA fragmentation, leading to death (Mazzoni and Falcone, 2008). FITC-VAD-FMK has an ability to penetrate the cell membrane and bind caspase to become fluorescent to be irreversibly activated by caspase (Shirazi and Kontoyiannis, 2013). It is used to monitor the caspase activation in this assay. Figure 5 shows that, on one hand, there were much fewer cells in the combined group compared with other groups. On the other hand, the combination group showed bright green fluorescence. In contrast, little fluorescence was observed in the control group and the single drug groups. Furthermore, the ratio of fluorescence cells in the combination group was much higher than in other groups as illustrated in Figure 6. The results demonstrated that the drug combination activated intracellular metacaspases and then induced the fungal cell death.

## Discussion

The invasive fungal infections remain a challenging problem. Current treatment for *C. albicans* infections mainly rely on azoles and echinocandins (Pristov and Ghannoum, 2019). The limited therapeutic option as well as the emergence of drug resistance is a matter of concern owing to the growing number of patients suffering from fungal infections (Hendrickson et al., 2019; Prasad et al., 2019). Hence, new therapeutic strategies are urgently needed. Several compounds with no antifungal effects were studied and exhibited an ability to enhance the activity of antifungal drugs, such as phytocompounds, calcium channel blockers, and compounds disrupting ion homeostasis (Liu et al., 2016; Zida et al., 2017; Li et al., 2018; Touil et al., 2018).

In *C. albicans*, histone deacetylases (HDACs) are essential components in the process of histone acetylation modification related to nucleosome assembly and remodeling pathways and play roles in gene transcription as well as DNA replication and repair (Li et al., 2015; Garnaud et al., 2016). Lysine deacetylases (KDACs) can also regulate heat shock protein 90 (Hsp90) acetylation (Gong et al., 2017). The inhibition of HDACs could ruin the growth and virulence of *C. albicans* and some HDAC inhibitors have shown antifungal activity combined with FLC *in vitro* (Garnaud et al., 2016). Geldanamycin (GA) is a specific inhibitor of Hsp90, and the combination of GA and FLC was synergistic against *C. albicans* strains (Zhang et al., 2013; Mahmoudi et al., 2019). Panobinostat is a pan-HDAC inhibitor. In this study, we tested the interaction of panobinostat combined with FLC against *C. albicans* both *in vitro* and *in vivo*.

Firstly, we examined the effects of panobinostat combined with FLC against both FLC-sensitive and FLC-resistant planktonic *C. albicans* cells. The results demonstrated that panobinostat displayed synergy with FLC against planktonic resistant *C. albicans* cells. The MIC of panobinostat reduced from 128 to 0.5–2 μg/ml, the MIC of FLC reduced from ≥512 to 0.25–0.5 μg/ml, and the FICI value was 0.0024–0.0166. Although there was no synergism when panobinostat was combined with FLC against planktonic FLC-sensitive strains (FICI > 0.5), the MIC of panobinostat notably decreased from 128 to 2–4 μg/ml and the MIC of FLC decreased from 0.25–1 to 0.25–0.5 μg/ml. Besides, the combination of panobinostat and FLC also showed antifungal effects against early-stage (≤8 h) biofilms regardless if it was FLC-sensitive or FLC-resistant *C. albicans.* The sMIC of FLC decreased from >1024 to 0.25–2 μg/ml and the sMIC of panobinostat could decrease from >128 to 0.5–8 μg/ml (FICI < 0.5). Meanwhile, the combination could notably reduce the biofilm formation compared with FLC alone as shown in Figure 1.

*Galleria mellonella* was selected for exploring *in vivo* antifungal activity of panobinostat and FLC. The doses of FLC and panobinostat were converted and explored according to the treatment dosage of human infections. As Figure 2 shows, the survival rate of the combination of panobinostat and FLC was 80%, which was much higher than that of fluconazole alone (35%) and the control group (10%), indicating that the combined treatment significantly prolonged the rate of survival of *G. mellonella*.

In addition, the mechanisms of drug synergism were explored in this study. It is known that biofilms are crucial for the development of candidiasis. The hyphal growth and the overexpression of drug efflux pumps are both major mechanisms of drug resistance (Nobile and Johnson, 2015). According to a series of previous researches, some drugs had synergistic effects with FLC against *C. albicans* strains because the combination could inhibit adhesion, inhibit hyphal formation, and suppress drug efflux (Gong et al., 2019b; Li Y. et al., 2019; Dwivedi et al., 2020). We previously studied the azole resistance of the strains in this study that was associated with overexpression of efflux pumps and high production of biofilms (Li et al., 2016; Lu et al., 2018; Gong et al., 2019b). Based on these studies, we first measured the effects of the drug combination on hyphal growth. As Figure 3 shows, after 4 h of incubation, the filamentation of the panobinostat–FLC combination was shorter and looser than that of the other three groups. The results revealed that the drug combination therapy could significantly inhibit *C. albicans* yeast–hyphae transition. This is only a kind of image analysis, and the hyphal growth could not be efficiently quantified at present.

As mentioned previously, panobinostat showed synergistic antifungal effects with FLC against early-stage biofilm formation, and efflux pump overexpression has been proven to have a powerful effect on FLC early resistance in *Candida* biofilms (Mukherjee et al., 2003). Hence, we also explored the effects of drug combination on drug efflux pumps. However, the result of this study indicated that the combination between panobinostat and FLC had no effect on the drug efflux pumps (*p* > 0.05); it may be associated with other factors such as cells’ ability of adherence or others that need to be further explored.

Caspases are cysteine proteases with specificity for aspartic acid residues in their substrates (Green and Llambi, 2015). The activation of caspase can interrupt DNA replication and DNA repair leading to apoptosis, which is a normal cell suicide program that is highly conserved among all species (Madeo et al., 2009). Inhibiting Hsp90 reduces apoptosis in *C. albicans*. It was demonstrated that GA could synergistically enhance tumor necrosis factor alpha (TNFα)- and TNF-related apoptosis-inducing ligand–triggered apoptosis by increasing the activation of caspase cascades in human malignant tumor cells (Bai et al., 2005). In *C. albicans*, metacaspase is the homologous form of caspase. Drug combination could also activate metacaspase associated with apoptosis in *C. albicans* (Kim et al., 2019; Li Y. et al., 2019). FITC-VAD-FMK *in situ* marker (Sigma), which is a pan-caspase inhibitor and fluoresces green when it binds to active metacaspases, was used to detect the activation of metacaspases in this study (Shirazi and Kontoyiannis, 2013). As illustrated in Figure 5, the cells under the treatment of panobinostat and FLC exhibited much brighter green fluorescence and much fewer total numbers compared with the other three groups. We also randomly selected 100 cells in the visual field and counted the number of fluorescence cells. There was a higher number of fluorescent cells in the combined group were much more than other groups (Figure 6). The results suggested that the combination of panobinostat and FLC might lead to metacaspase activation in *C. albicans*.

## Conclusion

In summary, panobinostat showed synergism with FLC against both resistant planktonic *C. albicans* and biofilms. Moreover, panobinostat plus FLC prolonged the survival rate of *G. mellonella* larvae infected with *C. albicans*. Mechanism studies elucidated that the synergism between panobinostat and FLC could inhibit the hyphal growth and activation of metacaspase. These findings might provide insights into overcoming the fungal resistance, and we hope this study could be helpful for novel antifungal drug development.

## Data Availability Statement

The raw data supporting the conclusions of this article will be made available by the authors, without undue reservation.

## Author Contributions

SS and SJS designed the experiments and wrote the manuscript. SS and XC performed the experiments. All authors analyzed the data and approved the manuscript for publication.

## Conflict of Interest

The authors declare that the research was conducted in the absence of any commercial or financial relationships that could be construed as a potential conflict of interest.
